# Esophageal Cancer Radiotherapy Dose Escalation Meta Regression Commentary: “High *vs*. Low Radiation Dose of Concurrent Chemoradiotherapy for Esophageal Carcinoma With Modern Radiotherapy Techniques: A Meta-Analysis”

**DOI:** 10.3389/fonc.2021.700300

**Published:** 2021-07-14

**Authors:** Ronald Chow, Michael Lock, Sangjune Laurence Lee, Simon S. Lo, Charles B. Simone

**Affiliations:** ^1^ New York Proton Center, Memorial Sloan Kettering Cancer Center, New York, NY, United States; ^2^ London Regional Cancer Program, London Health Sciences Centre, Schulich School of Medicine & Dentistry, University of Western Ontario, London, ON, Canada; ^3^ Tom Baker Cancer Centre, Alberta Health Services, Cummings School of Medicine, University of Calgary, Calgary, AB, Canada; ^4^ Department of Radiation Oncology, University of Washington School of Medicine, Seattle, WA, United States

**Keywords:** esophageal carcinoma, meta regression, squamous cell cancer (of the esophagus), adenocarcinoma, dose escalation

## Introduction

Esophageal cancer is one of the most common and lethal cancers in the world, with 600,000 cases and accounting for 544,000 cause-specific mortalities in 2020 (1). It is typically treated with definitive chemoradiotherapy or with trimodality therapy, but overall survival rates with both approaches remain dismal; the 5-year overall survival (OS) with chemoradiotherapy is only 10-20% (2, 3). Furthermore, high rates of local failure and distant metastases are reported. The search for improving our current management of these patients is urgently needed.

Several studies have, therefore, been initiated, assessing the role of dose escalation for patients receiving definitive radiotherapy (4–7). Success of dose escalation has varied. In particular, seminal trials such as INT 0123 (RTOG 94-05) investigated dose escalation from 50.4Gy to 64.8Gy and found no OS advantage with higher doses (8). Small sample size, confounding variables and limited statistical power may have limited meaningful conclusions, but impactful prospective dose escalation research thereafter seemed to have stalled.

Therefore, methodical meta-analyses are perhaps of greatest help to clinicians to address this question. Sun et al. recently conducted a systematic review and meta-analysis comparing high-dose radiotherapy to standard-dose radiotherapy in the setting of definitive concurrent chemoradiotherapy for esophageal cancer (9). With the pooled sample size across 12 studies and greater statistical power, they reported superior OS and local-regional control rates for patients receiving high-dose radiotherapy, and no difference in distant metastasis rate.

## Discussion

We commend the authors for a thorough and informative study that helps to inform radiation dosing for non-operable patients. However, there exists heterogeneity in their analyses that are worth commenting on and reanalyzing. This may or may not be accounted for by the degree of dose escalation in individual studies. Specifically, the magnitude of dose escalation may be an effect-modifier. To address this possible moderator variable, we conducted a meta-regression of study results, as identified by Sun et al. (9). Meta-regression is a meta-analytic method that specifically accounts for possible confounders to reveal the true effect of the variables of interest.

We included all 12 studies (4–7, 10–17) in our meta-regression. The difference in median dose of patients receiving high-dose and standard-dose radiotherapy was noted, per each study. Study data on OS, local-regional failure rate and distant metastasis rate were extracted, and cross-validated with that reported by Sun et al. (9). All studies reported on OS and were analyzed; stratified analyses by patient population (squamous cell carcinoma, and both squamous cell carcinoma and adenocarcinoma patients) were also conducted. Six studies (5, 6, 13–16) reported on local-regional failure and distant metastasis failure rates and were analyzed. A random-effects weighting was used for meta-regression when heterogeneity was high (I^2^ > 50); a fixed-effects weighting was used for low heterogeneity (I^2^ < 50). P-values less than 0.05 were considered statistically significant. All analyses were conducting using Stata 16.1 (StataCorp, College Station, TX, USA).

**Figure 1:**
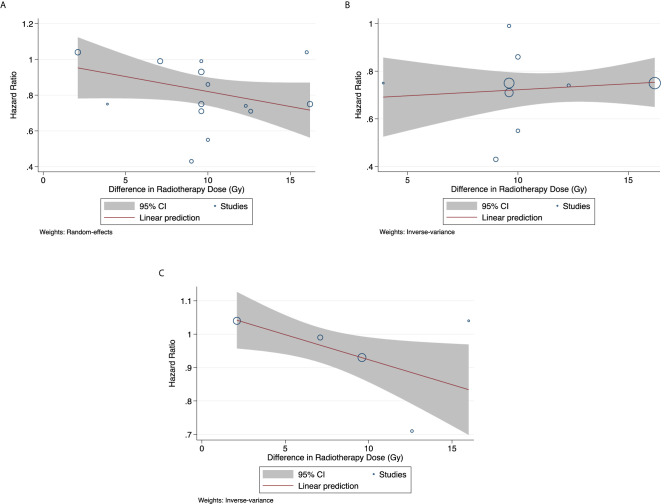
Overall Survival **(A)** For all studies (*p* = 0.104) **(B)** For studies on squamous cell carcinoma (*p* = 0.608) **(C)** For studies reporting on mixed population of squamous cell carcinoma and adenocarcinoma (*p* = 0.034).

There exists a trend for improved OS, with greater dose escalation (**Figure 1**; *p* = 0.104). Among studies only reporting on squamous cell carcinoma patients, OS did not improve with greater dose escalation (*p* = 0.608). In studies reporting on a mixed population of squamous cell carcinoma and adenocarcinoma patients, OS significantly improved with greater radiotherapy dosage (*p* = 0.034). Local failure rate and distant metastasis rate remain unchanged regardless of the degree of dose escalation varied (**Appendix 1**).

It is important to mention that in all analyses other than the OS analysis of all studies, there is only one study where dose escalation was in excess of 14 Gy. This sole datapoint, likely an influential point, makes it difficult to attain enough statistical power for this analysis. Nevertheless, this analysis suggests that dose escalation may be an effective strategy to improve the currently poor outcome of esophageal cancer patients and should be further explored.

Lastly, landmark trials such as the INT 0123 (RTOG 94-05) trial (8) that established lower doses of 50.4 Gy as standard have been criticized as less applicable to modern radiotherapy (18). Furthermore, 7 out of 11 patients who died in the high dose arm received 50.4 Gy or less. We eagerly await the results of the now-completed ARTDECO randomized phase III trial of dose escalation in a more modern cohort of esophageal cancer patients. Additionally, with the increasing use of advanced radiotherapy techniques such as intensity-modulated radiation therapy, proton therapy and MR-guided radiotherapy (19) today, the risk-benefit ratio of dose escalation may be further improved. Furthermore, with increasing consideration for using smaller radiation fields and/or omission of elective nodal irradiation for esophageal cancer (20), dose escalation may become safer and more widely utilized in the future.

In summary, we fully support prospective assessment of dose escalation for non-operable esophageal cancer, and the findings by Sun et al. (9) and our updated analysis in this commentary should be updated as additional data emerge, including studies using advanced radiation modalities and smaller radiation fields.

## Author Contributions

All authors listed have made a substantial, direct, and intellectual contribution to the work and approved it for publication.

## Funding

This research was funded, in part, through the NIH/NCI Cancer Center Support Grant P30 CA008748. 

## Conflict of Interest

The authors declare that the research was conducted in the absence of any commercial or financial relationships that could be construed as a potential conflict of interest.

## Supplementary Material

Supplementary Appendix 1
**(A) **Local-Regional Failure Rate (*p* = 0.426) **(B)** Distant Metastasis Rate (*p* = 0.837).

## References

[1] Sung H Ferlay J Siegel RL Laversanne M Soerjomataram I Jemal A. Global Cancer Statistics 2020: GLOBOCAN Estimates of Incidence and Mortality Worldwide for 36 Cancers in 185 Countries. CA: A Cancer J Clin (2021). 10.3322/caac.21660 33538338

[2] Cooper JS Guo MD Herskovic A Macdonald JS Martenson JA Jr. Al-Sarraf M. Chemoradiotherapy of Locally Advanced Esophageal Cancer: Long-Term Follow-Up of a Prospective Randomized Trial (RTOG 85-01). Radiat Ther Oncol Group JAMA (1999) 281:1623–7. 10.1001/jama.281.17.1623 10235156

[3] Yen YC Chang JH Lin WC Chiou JF Chang YC Chang CL. Effectiveness of Esophagectomy in Patients With Thoracic Esophageal Squamous Cell Carcinoma Receiving Definitive Radiotherapy or Concurrent Chemoradiotherapy Through Intensity-Modulated Radiation Therapy Techniques. Cancer (2017) 123:2043–53. 10.1002/cncr.30565 28152166

[4] Brower JV Chen S Bassetti MF Yu M Harari PM Ritter MA. Radiation Dose Escalation in Esophageal Cancer Revisited: A Contemporary Analysis of the National Cancer Data Base, 2004 to 2012. Int J Radiat Oncol Biol Phys (2016) 96:985–93. 10.1016/j.ijrobp.2016.08.016 27869098

[5] Clavier JB Antoni D Atlani D Ben Abdelghani M Schumacher C Salze P. Chimioradiothérapie Exclusive Pour Cancer De L’œsophage: Comparaison Entre 66Gy Et 50Gy, Une Étude Rétrospective. Cancer/Radiothérapie (2013) 17:221–8. 10.1016/j.canrad.2013.01.017 23684111

[6] He L Allen PK Potter A Wang J Chang JY Gomez DR. Re-Evaluating the Optimal Radiation Dose for Definitive Chemoradiotherapy for Esophageal Squamous Cell Carcinoma. J Thorac Oncol (2014) 9:1398–405. 10.1097/JTO.0000000000000267 25122435

[7] Li CC Fang HY Lin CY Shen WC Chien CR. Outcomes of Localized Esophageal Squamous Cell Carcinoma Patients Treated With Definitive Concurrent Chemoradiotherapy Using Either Standard or High Radiotherapy Dose: A Retrospective Study Controlling for Organ at Risk Dose. Anticancer Res (2019) 39:511–7. 10.21873/anticanres.13142 30591503

[8] Minsky BD Pajak TF Ginsberg RJ Pisansky TM Martenson J Komaki R. INT 0123 (Radiation Therapy Oncology Group 94-05) Phase III Trial of Combined-Modality Therapy for Esophageal Cancer: High-Dose *Versus* Standard-Dose Radiation Therapy. J Clin Oncol (2002) 20:1167–74. 10.1200/JCO.2002.20.5.1167 11870157

[9] Sun X Wang L Wang Y Kang J Jiang W Men Y. High *vs*. Low Radiation Dose of Concurrent Chemoradiotherapy for Esophageal Carcinoma With Modern Radiotherapy Techniques: A Meta-Analysis. Front Oncol (2020) 10:1222. 10.3389/fonc.2020.01222 32850362 PMC7418493

[10] Chang CL Tsai HC Lin WC Chang JH Hsu HL Chow JM. Dose Escalation Intensity-Modulated Radiotherapy-Based Concurrent Chemoradiotherapy is Effective for Advanced-Stage Thoracic Esophageal Squamous Cell Carcinoma. Radiother Oncol (2017) 125:73–9. 10.1016/j.radonc.2017.08.025 28923576

[11] Chen CY Li CC Chien CR. Does Higher Radiation Dose Lead to Better Outcome for Non-Operated Localized Esophageal Squamous Cell Carcinoma Patients Who Received Concurrent Chemoradiotherapy? A Population Based Propensity-Score Matched Analysis. Radiother Oncol (2016) 120:136–9. 10.1016/j.radonc.2016.04.042 27207358

[12] Deng Y Bian C Tao H Zhang H. Improved Survival With Higher Radiation Dose for Esophageal Squamous Cell Carcinoma Patients Treated With Definitive Chemoradiotherapy. Oncotarget (2017) 8:79662–9. 10.18632/oncotarget.19030 PMC5668079 29108346

[13] Ke TM Fong Y Lin LC Chien YW Yang CC Lin CH. Evaluating the Optimal Radiation Dose for Definitive Chemoradiotherapy for Esophageal Squamous Cell Carcinoma: A Single Institution Experience. Med (Baltimore) (2018) 97:e13214. 10.1097/MD.0000000000013214 PMC6257338 30431596

[14] Kim HJ Suh YG Lee YC Lee SK Shin SK Cho BC. Dose-Response Relationship Between Radiation Dose and Loco-Regional Control in Patients With Stage II-III Esophageal Cancer Treated With Definitive Chemoradiotherapy. Cancer Res Treat (2017) 49:669–77. 10.4143/crt.2016.354 PMC5512369 27737537

[15] Ren X Wang L Han C Ren L. Retrospective Analysis of Safety Profile of High-Dose Concurrent Chemoradiotherapy for Patients With Oesophageal Squamous Cell Carcinoma. Radiother Oncol (2018) 129:293–9. 10.1016/j.radonc.2018.09.006 30270099

[16] Zhang W Luo Y Wang X Han G Wang P Yuan W. Dose-Escalated Radiotherapy Improved Survival for Esophageal Cancer Patients With a Clinical Complete Response After Standard-Dose Radiotherapy With Concurrent Chemotherapy. Cancer Manag Res (2018) 10:2675–82. 10.2147/CMAR.S160909 PMC6097517 30147366

[17] Zhu WG Zhou K Yu CH Han JH Li T Chen XF. Efficacy Analysis of Simplified Intensity-Modulated Radiotherapy With High or Conventional Dose and Concurrent Chemotherapy for Patients With Neck and Upper Thoracic Esophageal Carcinoma. Asian Pac J Cancer Prev (2012) 13:803–7. 10.7314/APJCP.2012.13.3.803 22631652

[18] Simone CB 2nd. First Randomized Trial Supporting the Use of Proton Over Photon Chemoradiotherapy in Esophageal Cancer. J Clin Oncol (2020) 38:2952–5. 10.1200/JCO.20.01405 32706638

[19] Lee SL Bassetti M Meijer GJ Mook S. Review of MR-Guided Radiotherapy for Esophageal Cancer. Front Oncol (2021) 11:628009. 10.3389/fonc.2021.628009 PMC8019940 33828980

[20] Zhu H Rivin Del Campo E Ye J Simone CB Zhu Z Zhao W. Involved-Field Irradiation in Definitive Chemoradiotherapy for Locoregional Esophageal Squamous Cell Carcinoma: Results From the ESO-Shanghai 1 Trial. Int J Radiat Oncol Biol Phys (2021). 10.1016/j.ijrobp.2021.02.053 33677048

