# Supplementary material for: Esophageal Cancer Radiotherapy Dose Escalation Meta Regression Commentary: “High vs. Low Radiation Dose of Concurrent Chemoradiotherapy for Esophageal Carcinoma With Modern Radiotherapy Techniques: A Meta-Analysis”
Source: Front Oncol. 2021 Jul 14;11:700300. doi: 10.3389/fonc.2021.700300 (PMC8317968; doi:10.3389/fonc.2021.700300)
Supplement: Supplementary Appendix 1 — (A) Local-Regional Failure Rate (p = 0.426) (B) Distant Metastasis Rate (p = 0.837). [file DataSheet_1.docx]

**Appendix 1.1** Local-Regional Failure Rate (*p* = 0.426) **1.2** Distant Metastasis Rate (*p* = 0.837)

**1.1**

**1.2**
